# Use of Coffee Roasting By-Products (Coffee Silverskin) as Natural Preservative for Fresh-Cut Fennel Slices

**DOI:** 10.3390/foods14091493

**Published:** 2025-04-24

**Authors:** Miriam Arianna Boninsegna, Alessandra De Bruno, Corinne Giacondino, Amalia Piscopo, Giuseppe Crea, Valerio Chinè, Marco Poiana

**Affiliations:** 1 Department of AGRARIA, University Mediterranea of Reggio Calabria, Via dell’Università 25, 89124 Reggio Calabria, Italy; miriam.boninsegna@unirc.it (M.A.B.); corinne.giacondino@unirc.it (C.G.); mpoiana@unirc.it (M.P.); 2 Department of Human Sciences and Promotion of the Quality of Life, San Raffaele University, 00166 Rome, Italy; alessandra.debruno@uniroma5.it; 3 Caffè Mauro SpA Zona Industriale Snc, 89018 Villa San Giovanni, Italy; laboratorio@caffemauro.com (G.C.); vchine@caffemauro.com (V.C.)

**Keywords:** agri-food waste, food by-products, coffee silverskin, ready-to-eat vegetable, fresh-cut fennel slices, shelf-life, nutritional quality, sensorial quality

## Abstract

The coffee roasting by-product, coffee silverskin, represents a serious problem in environmental pollution. Still, it is also an interesting source of chemical compounds that can be recovered and used in the food industry to improve the physical, chemical, and sensory properties of a wide range of food products. This study aimed to evaluate, for the first time, the effect of the coffee silverskin extract (CSE), applied as a dipping treatment, in preserving the storage and the qualitative decay of fresh-cut fennel slices during 14 days of storage at 4 °C. The experimental plan evaluated two dipping solutions (5% and 10%) with coffee silverskin extract and compared them with a conventional dipping in 2% ascorbic acid and a control (water). The use of CSE in the dipping of fresh-cut fennel permitted an increase in the phenolic (chlorogenic and caffeic acids) content for up to 14 days, with good sensory acceptability and physico-chemical and microbiological characteristics. To date, no applications of CSE in this form have been reported, nor has any food by-product extract been investigated for the preservation of fresh-cut fennel, which makes this study a novel contribution to the development of sustainable treatments for minimally processed vegetables.

## 1. Introduction

Modern society faces a serious problem related to the sustainable management of by-products from food processing. It is imperative to find quick and effective solutions to encourage their reuse in view of the rapid transition toward an economy that complies with the goals proposed in the “2030 Agenda” in order to encourage zero-waste production as well as a green and circular economy [1,2]. Similarly, today’s consumers have increased the demand for natural additives to preserve the overall quality of “natural” and “sustainable” foods. These factors lead scientific research toward the recovery of bioactive compounds from industrial by-products and their use in food applications, such as for perishable vegetables, to prevent the rapid loss of quality during storage [3,4].

There is consistent evidence in the literature supporting the role of food by-products in extending the shelf-life of perishable fruits and vegetables by mitigating chemical, sensory, and microbiological spoilage. For instance, pomegranate peel extract has been shown to effectively delay chemical and sensory decay as well as inhibit pathogenic growth (*Listeria monocytogenes*) in fresh-cut pears, apples, and melons [5]. Additionally, it maintains the overall quality of ready-to-eat fruit salads [6] and even contributes to boosting Vitamin C content and preserving the texture and color of pears for up to 7 days of storage at 4 °C [7]. Similarly, grapefruit by-products have helped in ensuring the microbiological safety of ready-to-eat lettuce, while lemon by-product extract applications on melons [8] and carrots [9] also reduced weight and nutrient loss and sustained favorable chemical, physical, sensorial, and microbiological parameters for 12–14 days in cold storage. Other studies have reported that mango and citrus by-products presented an appreciable capacity in combating oxidative damage in fresh or processed potatoes [10,11]. Furthermore, other food by-products, such as banana [12], watermelon [13], and radish [14], have demonstrated a rich composition of bioactive molecules with antioxidant and antimicrobial potential, which contributes to food quality and safety.

The growing corpus of scientific research highlights the potential of bioactive compounds from food by-products as sustainable alternatives to conventional preservatives, thus laying the groundwork for further investigation. Despite the growing interest in this area, the functional properties and potential applications of many food by-products remain underexplored. In this context, coffee roasting by-products represent a promising yet largely untapped resource since, to date, they have not been thoroughly investigated, and their potential role in maintaining the quality and safety of fresh-cut vegetables remains to be elucidated.

In the last decade, the world’s coffee production grew from 8.5 million to 11 million tons, which is driven by a 29.4% increase between 2015 and 2023, thus reflecting growing consumer demand and the expansion of commercial coffee-based products [15,16]. Consequently, the intensification of coffee processing has led to a substantial accumulation of coffee silverskin (CS), a by-product exclusive to coffee roasting, with quantities ranging between 200,000 and 400,000 tons per year [17]. The disposal issues of CS in the environment constitute a profound pollution problem since its chemical composition, which is related to organic load, could be harmful to groundwater, soil microflora, and greenhouse gas production [18]. There is, therefore, an urgent need to find new sustainable strategies for its management and exploitation. Several studies have already indicated CS as a source of countless bioactive compounds with strong antioxidant activity, with chlorogenic and caffeic acid among the most representative ones [19,20,21]. The compounds present in CS were characterized by complex and varied chemical structures [22,23]; therefore, their recovery was a critical operation that required many attempts to identify the right balance between the method used and the extraction variables [24]. In recent years, the extraction process increasingly involved the use of food grade and green solvents, such as ethanol and water, since they enhance the recovery of valuable compounds from by-products, improving the sustainability of extraction and allowing for the direct use of extracts in food application; they are recognized GRAS (Generally Recognized as Safe) [24]. Among them, Costa et al. [23] reported the effectiveness of hydroalcoholic mixtures composed of ethanol and water to maximize the recovery of valuable compounds from CS. The bioactive compounds present in CS were recognized as being able to improve the characteristics of foods and, at the same time, help improve the health of the final consumer [25,26]. Therefore, CS powder and CS extracts have already been used for the formulation of various enriched foods such as drinks [27], cakes [28], cookies [29], yogurt [25], candies [16], and sausages [30]. Furthermore, the safety of CS was already reported; they present no carcinogen risk and very low or no presence of toxic substances such as ochratoxin A (OTA), pesticides, and polycyclic aromatic hydrocarbons (IPA) [25].

While other by-products were widely investigated to preserve the storage quality of fresh-cut vegetables [5,6,7,8,9,10,11,12,13,14], the effect of CS compounds on the physical, sensory, and microbiological characteristics is still poorly studied. Building on this premise, fennel (*Foeniculum vulgare* Mill. subsp. *vulgare*), a winter vegetable typical of the temperate Mediterranean regions—among which Italy is the largest producer in Europe [31]—represents a noteworthy example of a perishable food product. In the markets, fennel is generally sold in the whole form (“grumulo”) since the commercialization as fresh-cut fennel is limited by the characteristic rapid browning after cutting, which is due to the polyphenol oxidase (PPO) activity [32], the loss of chlorophylls a and b [33,34], and the rapid dehydration of tissues [35,36]. In addition, the minimal processes to which fresh-cut vegetables are subjected expose them to a greater risk of spoilage microorganism proliferation, causing rapid tissue deterioration and health dangers for consumers [37]. Visual appearance, firmness, and typical freshness taste are the main discriminants in consumers’ acceptance of fresh vegetables, particularly fennel. The loss of typical freshness aroma was mainly attributed to changes in organic acid content during storage, a normal process that is sped up in ready-to-eat vegetables due to stress resulting from cutting and the physiological responses associated with it (high respiration rate, weight loss, and sugar depletion) [32]. Several compounds, such as ascorbic and citric acid, which are used alone or in combination, have already been acclaimed as having inhibitory effects on enzymatic browning [38] and slowing down metabolic activity [32]. However, scientific research on strategies specifically targeting fresh-cut fennel slices remains limited, with few studies having explored effective approaches to preserving its overall quality and extend its shelf-life [31,32,39]. Capotorto et al. [31] indicated that a 0.5% ethanol solution delayed the quality loss in fresh-cut fennel, while 1% citric acid and 0.5% 4-hexylresorcinol determined a significant worsening, especially for the cut surface’s browning. Valentino et al. [39] further demonstrated that the application of active coating based on sodium caseinate also prevents the faster decay of fresh-cut fennel slices for up to 15 days of storage, although such edible coating alone was inefficient in counteracting PPO-mediated browning.

This study aimed to explore the novel use of coffee silverskin extract (CSE) through the formulation of a dipping solution for fresh-cut fennel slices to evaluate its effectiveness in preserving their chemical, physical, microbiological, and sensory characteristics. Furthermore, CSE was compared with an antioxidant treatment commonly used in the pretreatment of ready-to-eat vegetables (ascorbic acid) to assess its potential as a substitute for conventional preservatives. Nevertheless, to date, no studies have explored the use of food by-products, such as the CSE extract, as a dipping treatment for fresh-cut fennel. This could represent a sustainable and innovative solution for encouraging their reuse and replacement with synthetic additives and more complex storage techniques that are expensive to implement. Addressing the growing demand for natural and suitable preservation methods of foods, the preliminary results of this study introduce a new strategy for preserving fresh-cut vegetables, which is in line with the principles of the circular economy and also suggests a new life for the coffee roasting industry by-products.

## 2. Materials and Methods

### 2.1. Extraction of Bioactive Compounds from Coffee Silverskin

Coffee silverskin (CS) was supplied by the Mauro S.p.A. Coffee industry (Reggio Calabria, Italy) as a blend of Coffea Arabica and Coffea Canephora (variety Robusta) 50:50. CS was transferred to laboratory of Food Technologies of University “Mediterranea” of Reggio Calabria (Italy) and then submitted to the preliminary treatments: dehydration at 50 °C until 10% of moisture, grinding and homogenization. The extraction of bioactive compounds was performed at 60 °C for 60 min by maceration of the CS sample with a hydroalcoholic mixture (water: ethanol, 70:30, solvent: sample 10:1) The obtained extract (CSE) was thus centrifuged (NF 1200 R, Nuve, Ankara, Turkey) (6000 rpm, 10 min, 20 °C), filtered on paper (0.45 mm) and stored at −21 °C [16].

The chemical analyses of coffee silverskin extract were executed in triple and exhibited as means ± standard deviation.

#### Chemical Characterization of CSE

The total content of phenolic compounds (TPC) was detected by a Folin–Ciocalteu method reported by Boninsegna et al. [16]. In a test tube, 2.5 mL of Folin–Ciocalteu reagent (10% *v*/*v*), 0.3 mL of appropriately diluted (1:20) CSE, and 2 mL of Na_2_CO_3_ solution (7.5% *w*/*v*) were mixed in a test tube for 15 min at 45 °C. The mixture was let react for 30 min at room temperature before reading the absorbance by a double-beam UV spectrophotometer (Perkin-Elmer UV-Vis λ2, Waltham, MA, USA) at 765 nm. The reaction mixture without a sample was used as blank. Results were expressed as mg of gallic acid equivalents (GAE) L^−1^ of CSE using a calibration curve of gallic acid (2–10 mg L^−1^).

Total tannin contents (TTC) and total flavonoid contents (TFC) were determined according to Costa et al. [23]. The reaction mixture to quantify the TTC was prepared by mixing 2.5 mL of Folin–Ciocalteu reagent (10% *v*/*v*), 0.5 mL of CSE, and 2 mL of Na_2_CO_3_ solution (7.5% *w*/*v*), then, after the incubation of 2 h (dark room), the absorbance was recorded at 725 nm. For TFC, in a graduate test tube, 0.5 mL of diluted CSE (1:1) was mixed with 0.3 mL of 25% (*w*/*v*) NaNO_2,_ and after 10 min of incubation 0.3 mL of 10% (*w*/*v*) AlCl_3_. Then, 2 mL NaOH 1 M was inserted, and the reaction mixture was brought to a known volume of 10 mL with distilled water. The spectrophotometric determination was carried out by utilizing a wavelength of 510 nm.

The results of TTC were expressed as mg of Tannic acid equivalents (GAE) L^−1^ of CSE using the calibration curve of Tannic acid (1–20 mg L^−1^), while TFC was expressed as mg of epicatechin equivalents (ECE) L^−1^ of CSE by the use of calibration curve of epicatechin (1–200 mg L^−1^).

The quantification of chlorogenic and caffeic acids was performed according to an opportunely modified procedure proposed by Brzezińska et al. [40]. Briefly, 5 μL of diluted (1:10) CSE were injected into the UHPLC PLATIN blue system (Knauer, Berlin, Germany) equipped with a Knauer blue orchid C18 column (1.8 mm, 100 × 2 mm)and a binary pump, coupled with a PDA^−1^ (Photo Diode Array Detector) PLATINblue (Knauer, Berlin, Germany) 0.1% Formic acid (A) and methanol (B) were the elution solvents and the chromatographic separation was conducted at 30 °C at the conditions reported in Table 1. Caffeic and chlorogenic acids were detected respectively at 280 and 330 nm. Their quantification was determined by external standards, and the results were expressed as mg L^−1^ of CSE.

The multitarget approach consisting of the simultaneous use of the DPPH, ABTS, and FRAP assays was used in order to determine the total antioxidant activity of the CSE extract. Considering the complexity of the analyzed matrix and the different ways of acting of the antioxidant compounds content in it, the use of DPPH, ABTS, and FRAP allowed covering mechanisms of action linked to the ability to donate hydrogen atoms, electrons, and metal reduction thus restitution a complete measure of the antioxidant activity of CSE.

The ABTS, DPPH, and FRAP tests were performed using the methodology proposed by Boninsegna et al. [16]. The results were quantified using a Trolox calibration curve (2–30 mM L^−1^) and expressed as mM equivalent Trolox (TE) L^−1^ of CSE.

The presence of total bacteria (TBC), yeasts, and molds (L&M) in CSE was determined by the protocol described by Nolasco et al. [19] and expressed as Log10 colony-forming units (CFUs) mL^−1^ of CSE.

### 2.2. Fresh-Cut Fennel Processing

The fennel (*Foeniculum vulgare* Mill. subsp. *vulgare*) was supplied from a local market farm situated in Reggio Calabria (Italy) and transported to the FoodTec laboratory of the Mediterranea University of Reggio Calabria. Thereafter, an inspection was conducted to select them based on the absence of defects and similar size. The whole fennels were then washed and sanitized under a jet of cold water for 2 min and immersed in sodium hypochlorite solution (100 ppm) for 2 min. Then, the whole fennels were dried on steel grids for 15 min at room temperature.

Subsequently, the fennels were trimmed and cut into slices (1 cm thickness) perpendicularly to the longitudinal axis with appropriately sharpened tools to prevent damage to tissues [31].

**Figure 1:**
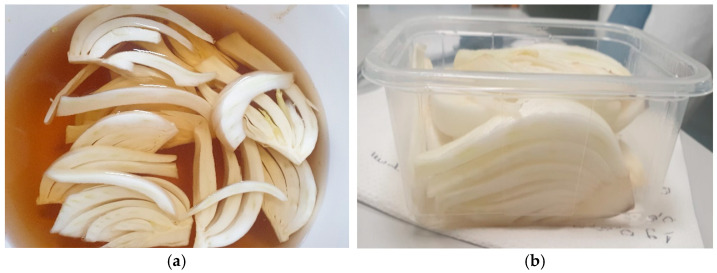
Fresh-cut fennel slices dipped in CSE treatment (**a**) and packaged in a PP tray (**b**).

In this study, CSE and AA were chosen as components of the dipping treatments for fresh-cut fennel sliced, and their concentrations were defined on the basis of previous scientific evidence [37,38,41,42]. In particular, as regards CSE, the focus was on its content of phenolic compounds, especially chlorogenic acid, known for its effectiveness as a preservative in fresh-cut vegetables subject to rapid browning [41,42], the major problem encountered in the storage of minimally processed fennel [31,32,39]. Therefore, the fennel slices were submitted for 5 min to three different dipping treatments: 2% (*v*/*v*) ascorbic acid (AA), 5% (*v*/*v*) CSE (CS5), and 10% (*v*/*v*) CSE (CS10) (Figure 1a). Fennel slices dipped in water were used as a control test (CTR).

The obtained samples were then dried (15 min), packaged (200 g) in a PP tray covered with a polyethylene terephthalate/polypropylene (PET/PP) film by a heat-sealing machine (VGP 25n, ORVED, Musile di Piave, VE, Italy) and stored at 4 °C for 14 days (Figure 1b).

The sensory attributes of fresh-cut fennel slices were evaluated at 0 and 14 days of storage, while the chemical, physical, and microbiological parameters were executed immediately after preparation (time 0 of storage) and after 3, 7, and 14 days of storage.

### 2.3. Qualitative Analyses of Fresh-Cut Fennel

#### 2.3.1. Sensory Analysis of Ready-to-Eat Fennel Slices

The sensory analysis was carried out following the reference standards in terms of selection criteria, training procedures, and verification of consistency of scores [43] in a sensory evaluation room complying with ISO 8589:2007 requirements [44].

Ten judges (aged between 21 and 42) were recruited from the organic staff of the University Mediterranea of Reggio Calabria (Italy). All judges had earlier expertise in sensory analysis across various food categories and were therefore pre-selected based on their backgrounds. Subsequently, training focused on the evaluation of sensory attributes specific to fresh-cut fennel based on descriptors reported in other studies on fennel minimally processed [31]. Weekly sessions (1.5 h) were held for one month to standardize the interpretation of each descriptor and align the panel on intensity scaling. By assessing the repeatability and reproducibility of the judges’ judgments, the consistency of the panel was assessed, complying with ISO 13299:2016 [43]. This process aims to ensure consistency and repeatability among assessors and to enhance the reliability of sensory data.

Before the sensory evaluations of fresh-cut fennel slices, all judges accepted the principles of Helsinki’s Declaration concerning the refrain from smoking, eating, and drinking (except water) before sensory evaluation to ensure unbiased evaluations. Then, the fennel slices were evaluated for visual, olfactory, and structural attributes as reported by Capotorto et al. [31], while the combination of visual, olfactory, and structural attributes decreed the score related to the total acceptability of fennel samples (overall acceptability). The panelists used a hedonistic scale from 0 to 9 to put the score. The limit acceptability was fixed at a 5 score. The descriptions of attributes and scores are reported in Table 2.

#### 2.3.2. Color and Textural Analysis of Ready-to-Eat Fennel Slices

The surface color parameters were determined by tristimulus colorimeter (Minolta CM-700d Spectrophotometer, Konica Minolta, Inc., Sakai, Osaka, Japan) with D65 illuminant using the CIE L* a* b* system reference, where L* was the lightness (0 black and 100 white), a* was red (positive value) or green (negative value) intensity and b* was the yellow (positive value) or blue (negative value) intensity of samples on the corresponding axis. The measurements were conducted at two points for each fennel segment on ten segments for each group.

The color differences (ΔE) after 14 days of storage were estimated by using the Equation (1).

∆E = [(∆L) 2 + (∆a) 2 + (∆b) 2] ½(1)

The structure analysis was performed using a TA-XT Plus Texture Analyzer (Stable Micro Systems Ltd., Godalming, UK). The hardness of the sample was evaluated by penetration test, following the method suggested by Rizzo et al. [45] with some modifications. A test was performed by compression, using a 2 mm cylindrical probe with a pre-test speed of 1.00 mm/S, test speed of 2.00 mm/S, post-test speed of 10.00 mm/S, distance of 4.0 mm, and trigger force of 5.0 g.

The crispiness of fennel samples was measured by the Volodkevich shear test. The use of a Volodkevich Bite Jaws consents to determine the consistency and crunchiness of samples to be assessed by simulating the action of the front incisors when biting the food. Therefore, previous studies suggest that the obtained data could return an instrumental evaluation comparable to sensorial analysis [46,47]. The following variables were used: test speed 2.00 mm/S and strain 50%. Hardness and crispness were expressed as the force (N) required to cause deformation and subsequent fracture of the sample. Exponent software 6.1.4.0 (Stable Micro Systems Ltd., Godalming, UK) was used for data gathering and curve integration.

#### 2.3.3. Total Soluble Solids, pH, Moisture, and Weight Loss 

The fresh-cut fennel (20 g) was homogenized with an Ultraturrax (T 25 digital, IKA, Staufen, Germany), centrifuged, and filtered by a paper filter (0.45 μm filter). Total Soluble solids (TSS, Bx°) and pH determinations were conducted on the obtained juices, following the method suggested by Escalona et al. [32], respectively by a digital refractometer (Atago Co, Tokyo, Japan) and a pH meter (pH 4, pH 7; Crison Basic 20, Crison Intrument, Barcellona, Spain) equipped with an ion-selective electrode.

Moisture content was estimated by placing 5 g of fennel sample inside a thermal balance (Sartorius Moisture Analyzer MA37, Sartorius Lab Instruments GmbH & Co. KG, Goettingen, Germany). The results were expressed on a percentage basis by using Equation (2) (AOAC 1994) [48]:

U.R. % = ((W0 − W1)/W0) × 100(2)

where W0 was the initial weight of the sample, and W1 was the final weight of the sample.

The weight loss was calculated as the difference between the initial and final weights of the fennel samples at different times of storage by the AOAC standard method (1984) [49], using Equation (3):

Weight loss (%) = ((Wi − Wt )/Wi ) × 100(3)

where Wi was the initial weight and Wt was the weight at time t.

#### 2.3.4. Quantification of Malic, Oxalic and Citric Acids

Oxalic, malic, and citric acids were detected by the experimental protocol described by Boninsegna et al. [50]. Moreover, 20 μL of fennel juice obtained as reported in Section 2.3.3, was injected in Knauer HPLC Smartline Pump 1000, equipped with a Knauer Smartline UV Detector 2600 and SYNERGY HYDRO-RP (250 mm × 4.6 mm i.d., 4 μm). Potassium phosphate 20 mM at pH 2.9 min was used in isocratic conditions at 22 °C at a flow rate of 0.7 mL. Ascorbic acid was recorded at 254 nm and other organic acids at 210 nm, with results expressed as mg of acid 100 g^−1^.

#### 2.3.5. Quantification of Caffeic and Chlorogenic Acids

Under ice, 10 g of fennel samples were homogenized by Ultraturrax with 20 mL of the hydroalcoholic mixture (methanol: water 80:20). Then, the mixture was centrifuged at 8000× *g* rpm (NF 1200R, Nüve, Ankara, Turkey) for 10 min at −2 °C, and filtered by a paper filter (0.45 μm). The obtained extract (5 µL) was submitted with some modifications to the chromatographic analysis described by Brzezińska et al. [40] for the determination of chlorogenic and caffeic acid concentrations (mg g^−1^).

#### 2.3.6. Microbiological Analysis

The microbial analysis was carried out according to Boninsegna et al. [50]. The microbiological suspensions were prepared by homogenizing 2 g of each sample with 20 mL of Ringer solution by Stomacher (BagMixer^®^ 400 P, Interscience, Saint-Nom-la-Bretèche, France) for 2 min. Subsequently, the obtained homogenates were serially diluted. Total aerobic mesophilic bacteria (TBC), yeast and molds, *Escherichia coli*, and *Listeria monocytogenes* were isolated using ready-to-use chromogenic plates (Compact Dry, R-Biopharm AG, Darmstadt, Germany).

### 2.4. Statistical Analysis

All analyses were carried out on three independent replicates to ensure the reliability and reproducibility of the results. Chemical and microbiological determinations were expressed as a mean ± standard deviation of three replicates. Textural and color determinations were expressed as means ± standard deviation of twenty measurements for each replicate of fennel samples. The sensory analysis was expressed as a mean ± standard deviation of the judges’ scores for each sensory attribute.

The analysis of variance (one-way ANOVA) was conducted by applying the post hoc Tukey test at *p* < 0.05 (SPSS software, version 15).

## 3. Results

### 3.1. Chemical Characterization of Coffee Silverskin Extract

The CSE exhibited a high content of antioxidant compounds related to phenolic (TPC), flavonoid (TFC), and tannin (TTC) content (Table 3). The microbiological analysis of CSE did not evidence bacterial, yeast, and mold contamination. From these results, CSE was considered useful as a food ingredient in the formulation of dipping solutions for fresh-cut fennel processing.

### 3.2. Quality Evaluations of Fresh-Cut Fennel Slices

#### 3.2.1. Sensory Analysis

Positive sensory evaluation is crucial to ensuring the success of marketable and consumer acceptability of ready-to-eat fruits and vegetables. 

**Figure 2:**
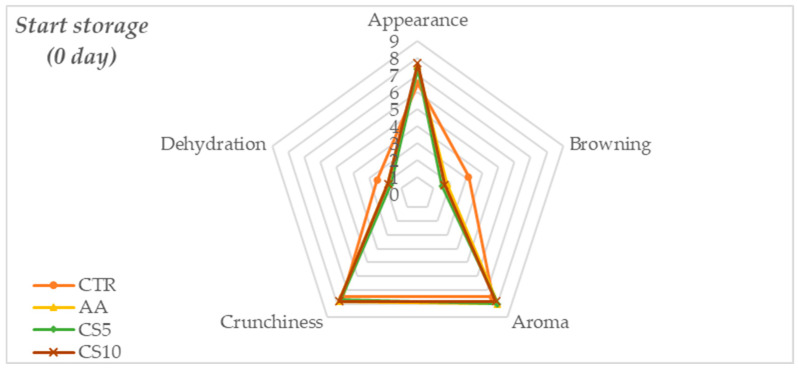
Spider plot of the means obtained for the individual sensory attributes at the beginning storage time of the fresh-cut fennel slices.

The results obtained by the sensory evaluation showed that at the beginning of storage (Figure 2), the fennel slices dipped with different pretreatments arose better than no treated samples with a high score obtained for CS10, followed by CS5 and AA, while the CTR sample showed a significantly lower score (*p* > 0.05), these trends were same for appearance, browning and dehydration parameters. In contrast, the aroma and the crispness did not show significant differences between treatments, indicating that initially, all samples retained these characteristics and were unchanged.

**Figure 3:**
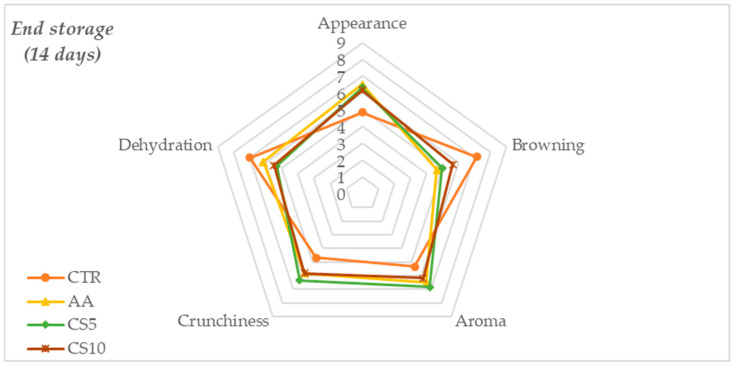
Spider plot of the means scores obtained for the individual sensory attributes at the end storage time of the fresh-cut fennel slices.

At the end of storage (14 days) (Figure 3), there was a general deterioration in sensory quality, with more marked differences between treatments (*p* > 0.01). In particular, the appearance worsened in all samples, but CS10 maintained the best score, followed by CS5 and AA. The other parameters related to browning, aroma, crunchiness, and dehydration also showed a similar trend, suggesting a dependence between the variation of sensory characteristics over time and the treatment applied. The CTR sample showed significantly more marked deterioration than samples dipped in a different pretreatment.

**Figure 4:**
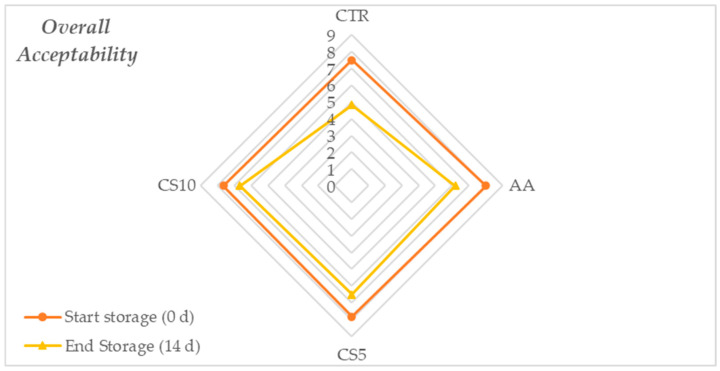
Spider plot of the mean scores obtained for the individual sensory attributes at the end of storage time of the fresh-cut fennel slices.

Overall acceptability scores (Figure 4) followed individual sensory trends. Initially, quality was uniform across treatments, but a decline occurred over time. CS5 and CS10 maintained better appearance, crispness, and aroma, outperforming AA, while CTR showed significant deterioration, falling below the acceptability threshold (score 5).

#### 3.2.2. Color and Textural Analysis

The variation of the L*, a*, and b* parameters was used to estimate the enzymatic browning and consumer acceptability of fresh-cut vegetables after the cutting operations.

L* values for all monitoring times reveal that there was an increasing loss in the CTR sample during storage, while the maintenance of values was found in the CS5 sample, where no significant (*p* < 0.05) difference was revealed up to 14 days of storage.

As shown in Table 4, CS5 and CS10 after the dipping treatment (0 days of storage) showed an increase in a* value than CTR and AA due to the pigmentation of the extract obtained from coffee by-products. However, a gradual increase in the a* value, from negative to positive values, was detected in CTR and AA and not in CSE and CS10, indicating the color change toward browning during storage. b* values did not show significant variations during the whole storage period in all the fresh-cut fennel samples.

From ∆E value elaboration, it was also confirmed that the most evident chromatic changes during storage were observed on CTR and AA, in the trend of CTR > AA > CS5 > CS 10 with values from 7.09 to 4.96.

**Figure 5:**
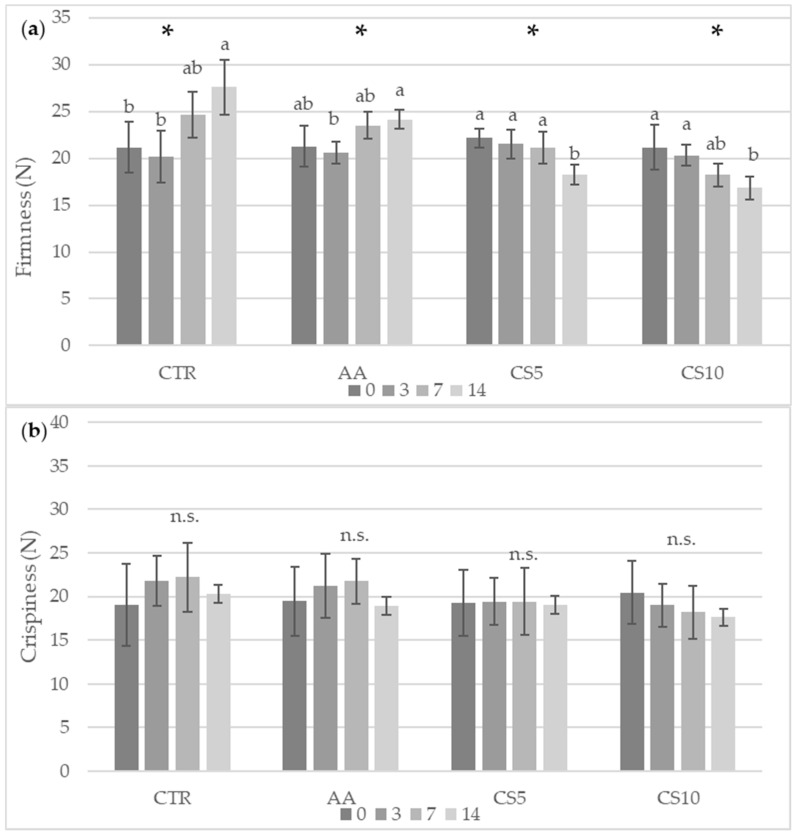
Textural parameter of fresh-cut fennel slices during the storage period for firmness (**a**) and crispiness (**b**). All results are shown as a mean ± standard deviation of replicates (*n* = 3). Small letters show significant differences as assessed by Tukey’s post hoc test. Abbreviations: *, significance at *p* < 0.05; n.s., not significant; CTR, control samples, AA, ascorbic acid dipping, CS5, coffee silverskin extract dipping (5%), CS10 coffee silverskin extract dipping (10%).

Regarding the firmness parameter (Figure 5), significant differences were found in the CTR and AA samples already after 7 days of storage, which showed a progressive hardening. CS5 and CS10 maintained instead in the same period their textural properties and tended to be less firm (<20 N) at the end of storage (Figure 5a). All the samples did not differ from each other during storage for the crispiness parameter without showing statistically significant differences between CSE treatments at different concentrations (Figure 5b).

#### 3.2.3. Total Soluble Solids, pH, Moisture and Weight Loss of Fresh-Cut Fennel Samples

**Figure 6:**
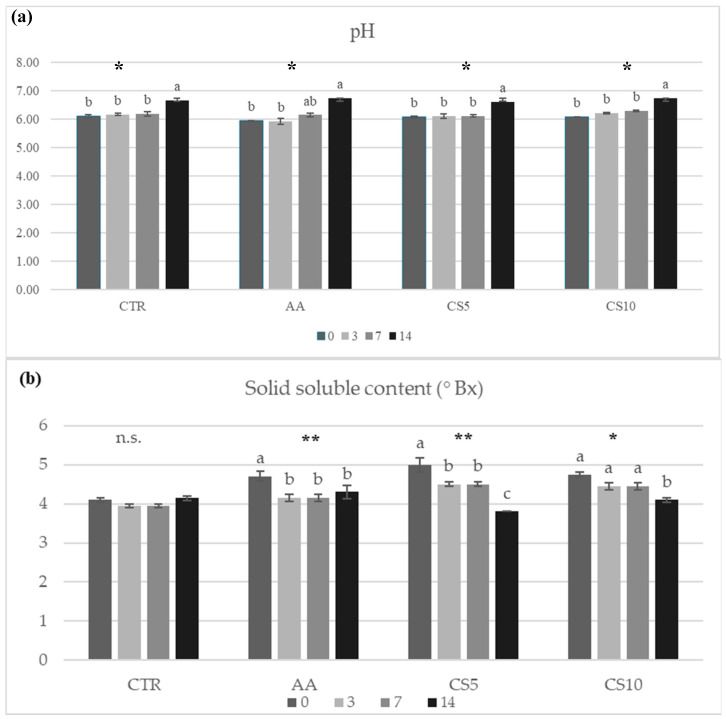
pH values (**a**) and solid soluble content (**b**) of fresh-cut fennel slices during the storage period. All results are shown as a mean ± standard deviation of replicates (*n* = 3). Small letters show significant differences as assessed by Tukey’s post hoc test, *p* < 0.05. Abbreviations: **, significance at *p* < 0.01; *, significance at *p* < 0.05; n.s., not significant; CTR, control samples, AA, ascorbic acid dipping, CS5, coffee silverskin extract dipping (5%), CS10 coffee silverskin extract dipping (10%).

Immediately after immersion in dipping solutions, a decrease in pH was found in sample AA (5.93), while CTR, CS5, and CS10 did not show differences with values around 6.1 (Figure 6a). These experimental data showed the pH value dependence with the immersion solution used. However, during the storage of fresh-cut fennel slices, no significant variations were found up to 7 days of storage, while a rise in pH value was found after 14 days of storage, an index of qualitative decay of fresh-cut vegetable, analyzed linked to the loss of organic acids characterizing the chemical composition of fennel. Total soluble solids in samples treated with dipping were higher than CTR samples at the start of storage and then tended to decrease up to 14 days, particularly in CS5 and CS10 (Figure 6b).

The moisture content of samples tended to decrease, particularly in the CTR sample (from 96.98 to 92.58 during storage), while it maintained similar percentages in AA, CS5, and CS10 (about 95.90).

**Figure 7:**
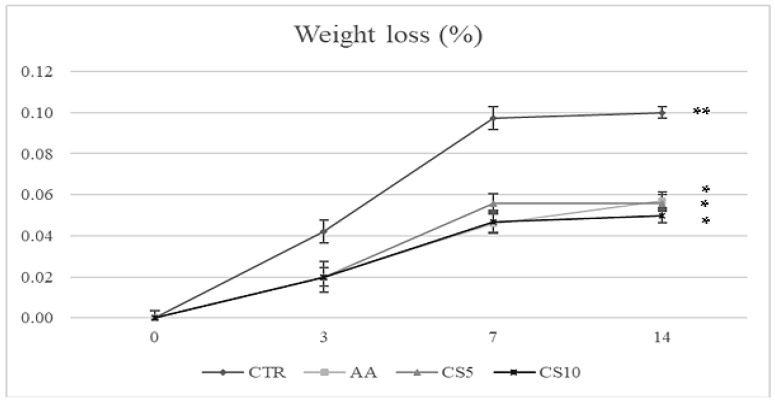
Weight loss of fresh-cut fennel slices during the storage period. All results are shown as a mean ± standard deviation of replicates (*n* = 3). Significant differences as assessed by Tukey’s post hoc test, *p* < 0.05. Abbreviations: **, significance at *p* < 0.01; *, significance at *p* < 0.05; CTR, control samples; AA, ascorbic acid dipping; CS5, coffee silverskin extract dipping (5%); CS10 coffee silverskin extract dipping (10%).

During storage, a gradual weight loss was found in all samples (Figure 7), with higher development in CTR than in the other samples.

This phenomenon is mainly due to a slowdown of the metabolic activities discharged by dipping treatments. In particular, a lower weight loss was found in CS10, followed by CS5 and AA, suggesting a greater maintenance of sensory and structural characteristics, as confirmed by the previous analyses.

#### 3.2.4. Quantification of Malic, Oxalic, and Citric Acid

The results shown in Table 5 suggested a marked dependence between the quantity and trends of organic acids and the dipping treatment to which the fennel slices were subjected. A significant dose-dependent effect (*p* < 0.05) was also recorded for fennel slices dipped in both CSE concentrations. A general increase was found at the beginning of storage for oxalic and malic acids, following the trend CS10 > CS5 > AA > CTR, while the citric acid in AA samples was similar to the CTR sample and significantly lower (*p* < 0.01) than the CS5 and CS10 samples. These trends were maintained during storage, up to 21 days, with levels of malic and oxalic acid remaining consistent, although with some fluctuations, while for citric acid, a more marked variation was observed during storage, especially in the CTR and AA samples. The trends observed in this study suggested a different sensitivity in the response of organic acid content and its maintenance over time to the treatment used.

#### 3.2.5. Quantification of Chlorogenic and Caffeic Acids

**Figure 8:**
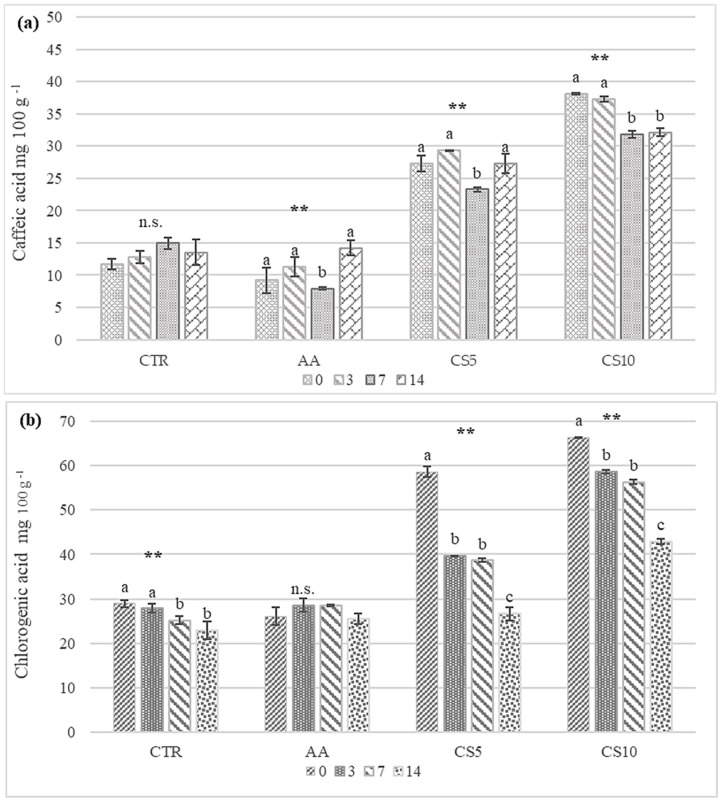
Trends of Caffeic (**a**) and Chlorogeic (**b**) Acids during the storage of fresh-cut fennel slices. All results are shown as a mean ± standard deviation of replicates (*n* = 3). Small letters show significant differences as assessed by Tukey’s post hoc test, *p* < 0.05. Abbreviations: **, significance at *p* < 0.01; n.s., not significant; CTR, control samples, AA, ascorbic acid dipping, CS5, coffee silverskin extract dipping (5%), CS10 coffee silverskin extract dipping (10%).

The trend of chlorogenic and caffeic acid showed that the dipping treatment with 5–10% CSE significantly increased (*p* < 0.01) their presence in slices of fresh-cut fennel, and this tendency was found throughout the storage (Figure 8). In particular, CS10 showed the highest content of these phenolic acids up to 14 days of storage.

The observed general reduction of chlorogenic acid was due to physiological processes mediated by key enzymes such as Polyphenoloxidsase (PPO) [4]. However, the high amounts of chlorogenic and caffeic acid during storage suggested that immersion in CSE may lead to a significant increase in these valuable compounds involved not only in preventing the rapid decay of vegetables and fruits but also in processes that lead to a positive effect on the health of consumers.

#### 3.2.6. Microbiological Analysis

During the 14 days of storage, all samples showed gradual growth of TBC (Table 6). At the end of shelf life, the lowest values were obtained from CS10, followed by CS5. This suggests a dose-dependent CSE effect. About yeasts, no statistical differences were found up to 14 days of storage; these microorganisms increased during storage in all samples. No molds were detected in all samples analyzed until the end of storage. In all samples, *Listeria monocytogenes* and *Escherichia coli* were not detected.

## 4. Discussion

The valorization of food by-products through their reuse in the food processing cycle represents today both a challenge and the greatest opportunity with which the food sector interfaces [2,3]. In this scenario, finding a collocation for the by-products resulting from the coffee roasting process is still a little explored but offers important advantages, especially related to its chemical composition and the potential related to it in food production enrichment [17,19,21]. For the recovery of bioactive compounds from by-products to be efficient, predisposing conditions must be used (time, temperature, solvent) that allow for the preservation of their structural integrity and activity. The first phase of this study was dedicated to extracting antioxidant compounds from CS to obtain an extract useful for preserving highly perishable vegetables, as has already been done for other food by-products [4]. Special focus was given to the phenolic profile, especially chlorogenic acid content, since it was identified as an effective ally in preserving the physical, chemical, and organoleptic properties of fresh-cut vegetables, especially those prone to faster cut surfaces’ browning [41,42], main problem detected in minimally processed fennel [31,32,39]. The chemical characterization of CSE obtained in this study showed a high concentration of chlorogenic acid (33.99 mg 100 mL^−1^) and significant amounts of polyphenols, flavonoids, and tannins, all of which contributed to its high antioxidant activity, as proved by the DPPH, ABTS, and FRAP assays (Table 3). The stereochemistry of the compounds in CS and the slow diffusion that takes place during maceration were the basis for obtaining high extraction yields’ of valuable compounds with high antioxidant activity [51]; this was aided by a suitable extraction temperature and time, which allowed the diffusion rate of compounds in the solvent to be increased without damaging them [52,53]. In particular, the synergy between the extraction method, the variables used during the process (temperature and time), and the extraction solvent (30% EtOH) allowed the recovery of valuable compounds, which proved to be indispensable in this study, in supporting the quality characteristics of fresh-cut fennel slices up to 14 days of storage at 4 °C.

Extracts derived from food by-products were already noted for their ability to preserve the quality characteristics of fresh-cut fruits and vegetables [16]. Also, in this pioneering study, the effects of dipping solutions with CSE and the interactions between the compounds in it to counteract the rapid decay of fennels were particularly appreciable concerning the preservation of the quality index related to sensorial, physical-chemical, and microbiological aspects.

Sensory perception, resulting from sensory evaluations, showed that immediately after treatment, no statistically significant differences in overall acceptability were found. These results were to be considered satisfactory as they suggested that CSE does not alter the sensory characteristics of fresh-cut fennel slices compared to conventional treatment with ascorbic acid (AA) and water (CTR). However, during the storage period, a gradual decay of sensory parameters was recorded for all samples, with a marked deterioration for CTR followed by AA and better maintenance of all sensory attributes found in CSE samples (Figure 2, Figure 3 and Figure 4). The score of overall acceptability recorded at the end of storage revealed that CS5 and CS10 samples differed significantly from CTR and slice samples treated with conventional dipping of ascorbic acid (AA) for better maintenance of appearance, freshness, aroma, and lower dehydration of the tissues, notably for CS10. The sensory stability over time was therefore significantly dependent on the treatment and, in the case of CSE samples, the higher scores compared to the other samples tested were due to the synergy between a higher concentration of organic acids [54,55], the action of chlorogenic acid [56] and the content of macronutrients [17] that contributed to the maintenance of aroma, texture and to counteract browning.

The common browning in fresh-cut vegetables is mainly due to complex enzymatic reactions related to cutting operations [57]. For this reason, especially in the ready-to-eat fennel, the difficult challenge is to find pretreatment, packaging, and storage strategies to avoid this happening [31,32,39,58,59,60]. The results of the colorimetric analysis showed a better action of CSE than AA in counteracting the browning of the cutting surface (Table 4). A prolonged reduction in lightness (L*) was observed in the storage time of CTR samples while the treated fennels were similar to each other, suggesting the dependence between L* and the applied dipping. However, during storage, a gradual increase in a* parameter, index of browning, was observed in CTR and AA fennels, while in CS5 and CS10, no significant differences were found. The values obtained for ∆E, a parameter used to compare instrumental color data with visual consumer acceptability, also confirmed the sensory and colorimetric evaluations. Samples CS5 and CS10 showed values ≤5, indicating colorimetric changes not perceptible by the human eye during storage [57]. Ascorbic acid was already recognized as an effective regulating effect on the oxidative metabolism of fresh-cut vegetables by acting directly on the structure of polyphenoloxidase (PPO), a key enzyme in the browning process, determining a change in structural conformation due to the chelation of the copper ions at the active site of the PPO [61]. It also limits the oxidation of total phenols by increasing the activity of antioxidant enzymes such as ascorbate peroxidase (APX) and inhibiting those pro-oxidant enzymes such as polyphenoloxidase (PPO) and peroxidase (POD). While the ascorbic acid’s anti-browning activity was already widely discussed for various fresh-cut vegetable categories [61,62,63], the effect of CSE remains unexplored. The anti-browning effect of CSE dipping solutions encountered by the process of experimental data was due both to the hydroalcoholic nature of the extract [31,64] and the high concentration of chlorogenic acid, which led to a rearrangement of the PPO secondary structure and a significant decrease in browning and oxidation products as already reported by Cheng and colleagues on fresh-cut potatoes [46]. Indeed, the presence of CSE in treated fennel slices was confirmed by higher levels of chlorogenic acid, the phenolic acid predominating in the coffee roasting by-products [65], suggesting clear dose-dependent effects in CS5 and CS10 compared to CTR and AA (Figure 8) in the maintenance of color. CSE, thus, proved to be an effective solution to countering the phenomenon of browning in minimally processed fennel, ensuring better maintenance of quality parameters over time compared to other approaches such as edible coating activated with propyl gallate [59] and preservation under modified atmosphere [60].

The potential of a fresh-cut vegetable to attract the final consumer also depends on the maintenance of firmness, which is an indicator of freshness over time [4]. However, the loss of firmness in vegetables is a natural phenomenon that, in fresh-cut fruits and vegetables, is aided by a multitude of biochemical and physiological variations that occur in response to wounding stress [66]. The fresh-cut vegetables showed an increase in the rate of respiration, transpiration, consumption of energy reserves (saccharides, nutrients, organic acids), rapid weight loss, as well as the activation pool of enzymes, which act on the cell wall, causing the reduction of intercellular adhesion, loss of turgor and the subsequent disintegration [66,67,68]. In other cases, the cut response can determine the activation of phenylpropanoid metabolism, which leads to the production of lignin from phenolic compounds by phenylalanine ammonia−lyase (PAL), determining the stiffening of vegetable tissues [69]. Preventing these damages represented the basis of good conservation of fresh-cut vegetables. In this context, the application of extracts from food by-products, such as lemon, grape, pomegranate, and olive, was tested in various categories of vegetables in the form of a pretreatment solution [70] or incorporated into edible coatings [71], yielding satisfactory results. However, there is currently a lack of scientific evidence on the use of food by-products, such as CSE, to preserve the storage quality of fresh-cut fennel slices. The firmness parameter (Figure 5) found in this study showed significant differences in the samples treated after 7 days of storage. CTR and AA exhibited progressive hardening due to moisture loss and weight loss (Figure 7), which was facilitated by the action of lignification mediated by PAL. In contrast, in CS5 and CS10 samples, better maintenance of firmness was observed for up to 7 days without significant differences between the CSE treatments; thereafter, enzymatic activity trigged by cutting-induced metabolic stress, enzymatic decompartmentalization and subsequent action on the cell wall led to a decrease in this parameter [70,71]. These results suggested a dependence between the treatment and the type of metabolic response to which the fruit and vegetables go after being subjected to cutting stress, denoting the activation of several enzymatic pools, already reported by Chen et al. [4] and Asrey et al. [72]. In accordance with the above, the analysis carried out by the variation of total soluble solids and pH (Figure 6) also confirmed the slowing down of metabolic activity and the better maintenance of CSE-treated fresh-cut fennel slices, with the values recorded within the ranges of 4.0–8.0 and 5.5–6.5 respectively, consistent with previous studies concerning the chemical quality of fennel [73]. The values of pH increase and decrease in SST are attributable to metabolic activities that occur in vegetables subjected to cutting operations, and their variations are directly proportional to the intense metabolic activity causing the rapid deterioration [71]. Experimental data from CTR samples followed by AA samples indicated a natural senescence process observed in vegetables [67,68], consistent with results in broccoli [74], cucumbers [68], and mushrooms [75] undergoing cutting and storage. Conversely, the response of the fennel to the CSE immersion treatment suggested the presence of additional valuable compounds in the extract, which can significantly slow down metabolic processes and improve the preservation of quality attributes during storage. Nzekoue et al. [17] and Franca et al. [76] reported CS as a rich source of macrominerals, particularly calcium, which is widely used in the treatment of fresh-cut vegetables to increase their textural properties and slow physiological responses following injury damage. The dipping of the fennel slices in CSE determined the interaction between the cell wall’s pectin and the calcium ions, favoring the formation of a fortified network around the cutting surface and thus allowing the maintenance of the characteristics of original freshness by slowing down the degradation due to hastened metabolic activity [77]. These positive textural effects of CSE were also reflected by a positive impact on the maintenance of other valuable nutrients and bioactive compounds, as confirmed by the analyses carried out regarding the quantification of organic acids and phenolic acids.

Fresh vegetables contain several bioactive compounds beneficial to human health, but preventing their loss during storage remains a difficult challenge. The use of natural extracts derived from food by-products helps to maintain the content of these compounds [78] and, in some cases, to increase their presence [50]. The results in Table 5 showed an increase in the concentration of the oxalic, malic, and citric acids in the fennel slices treated with CSE and a better retention of these over the storage time, while the AA sample showed better retention of malic and oxalic acids and a drastic reduction in citric acid compared to CS5, CS10, and CTR samples. Gong et al. [79], Erbaş [54], and Galani et al. [53] reported that the major causes of the variable tendency of organic acids during the storage of fruit and vegetables were attributed to the degradation of precursor compounds, oxidative stress, stress responses, interaction with microorganisms and storage conditions. In addition, also the quantification of phenolic acids most present in CSE (caffeic and chlorogenic) showed a dose-dependent increase and better retention in fennel slices treated with dipping solution CSE than others (Figure 8), notably for CS10. In this study, the trends of organic acids supported the sensory and physical analyses in explaining the better maintenance of parameters considered in CS samples since they were closely related to the better sensory attribute’s perception [32] as well as the better maintenance of textural and color parameters [38]. The trends observed for phenolic acids also helped to explain the anti-browning, sensorial, and textural response of fennel slices treated with CSE. Chlorogenic acid and caffeic acid were reported as powerful antioxidants that can delay enzymatic browning and sensory degradation, protecting the cell structure and helping to preserve color and aroma [56,80]. On the other hand, the high presence suggested that a dipping treatment, as proposed in this study, would allow enriching the chemical composition of fresh-cut fennel slices with valuable compounds allied to human health (protecting oxidative harm, carbonyl stress, accumulation of advanced glycation end products (AGEs) and prebiotic activity) [81,82,83].

Alongside the chemical, physical, and sensory parameters, microbiological quality is also a critical parameter in the storage of fresh-cut vegetable products, as it directly affects both shelf-life and finally, consumers’ safety [78]. Table 6 reveals that the dipping treatments of CS and AA led to a significant effect on mesophilic bacteria grown (TBC) from the 7 days of storage, while no molds or human bacteria pathogenic (*Listeria monocytogenes* and *Escherichia coli*) were detected during the entire storage period on all fresh-cut fennel slices. Regarding yeasts, no statistical differences were found up to 14 days of storage; these microorganisms increased during storage in all samples. The results suggested that all fennel slices treated with dipping solutions (AA, CS5, and CS10) were microbiologically safe for up to 14 days of storage, with better results obtained for CS5 and CS10. The slowing of microbial growth by ascorbic acid and other organic acids was already extensively documented in previous studies [30,84,85,86], while the effect on microbial flora by CSE, recorded in this study, was not yet investigated and can be due to the synergy of nature the hydroalcoholic nature of extract [87] and their chemical composition [88,89,90]. The biocidal and fungicidal activity of organic acids (malic and citric acids) and phenolic acids (chlorogenic and caffeic) has already been documented for gram-positive bacteria, gram-negative bacteria, and various molds such as *Pseudomonas* spp., *Escherichia coli*, *Salmonella* spp., *Listeria* spp., *Candida* spp., and *Aspergillus* spp. [91,92,93]. The action of these molecules is mainly due to the chelation mechanisms of metals, interaction with the cell membrane, and alteration of osmotic equilibrium that directly interferes with microbial metabolism [92,93]. The fennel slices immersed in CSE showed a significantly higher content of organic acids and phenolic acids than the other tests. Therefore, the enrichment in their chemical composition and the synergic action of these compounds were decisive in maintaining the proliferation of microorganisms, at levels deemed safe for the consumer [94], up to 14 days of storage.

## 5. Conclusions

This study highlights the potential of silverskin coffee extracts as a sustainable and functional alternative to conventional preservatives such as ascorbic acid, thus taking advantage of their rich composition in bioactive compounds. The results open new perspectives on the effectiveness of preserving the total quality and, at the same time, bring an improvement to bioactive compounds of fresh-cut fennel slices, offering a natural and ecological approach to food preservation. When compared to ascorbic acid treatments and untreated tests, the CSE samples (especially CS10) showed better maintenance of chemical, physical, microbiological, and sensorial characteristics. Furthermore, CS also leads to a dose-dependent increase in valuable substances such as the organic acids (oxalic, phenolic, and malic) and phenolic acids (caffeic and chlorogenic) directly involved in the slowing down of metabolic processes and complex reactions, which underlies better maintenance of the overall acceptability of fresh-cut fennel slices up to 14 days of storage.

These results were encouraging and represent a first step in the potential use of coffee silverskin hydroalcoholic extract in food applications and as a potential replacement of synthetic additives in maintaining chemical, physical, microbiological, and sensory characteristics of highly perishable and rapidly browning vegetables.

Despite the promising results observed in this study, it is important to note that, as far as we know, extracts from food by-products for improving the shelf life of fresh-cut fennel slices have not yet been tested, and CSE has not been previously used in dipping treatments for fresh-cut vegetables and fruits. Therefore, future research perspectives could focus on comparing the efficacy of CSE with other extracts derived from food by-products, further exploring its potential to preserve a wider range of highly perishable plant products.

The use of CSE, as proposed in this study, was a simple and eco-friendly strategy for upgrading food by-products derived from the coffee roasting process. This gives it new life in the food sector in view of the rapid transition toward a circular economy model.

## Figures and Tables

**Table 1:** Elution program used to detect chlorogenic and caffeic acid in coffee silverskin.

Time (min)	Eluent A (%)	Eluent B (%)	Flow Rate (mL/min)
Initial	98.00	2.00	0.40
3.00	80.00	20.00	0.40
9.00	50.00	50.00	0.40
14.00	50.00	50.00	0.40
16.00	80.00	20.00	0.40
18.00	95.00	5.00	0.40
20.00	95.00	5.00	0.40

**Table 2:** Description of attributes and corresponding scores used for sensory analysis of fresh-cut fennel slices.

Attribute	Description Attribute	Description Score
Visual		
Appearance	Evaluation related to the perception of fresh and freshly cut vegetables: Intensity of white and brightness, draining of surfaces, sheath and stem intact and free from defects.	0 Total absence of required visual attributes (not acceptable); 5 slight perceptions of (acceptability limit); 9 Presence of visual attributes like as fresh vegetable perception (excellent acceptability).
Browning	Browning on cutting surface, sheath, and stem	0 No browning (excellent acceptability); 5 Modest browning (acceptable limit); 9 Severe browning (not acceptable).
Olfactory		
Aroma	The aroma of freshly cut fennel associated with fresh and herbaceous	0 No presence (not acceptable); 5 Moderate Presence (limit acceptability); 9 Intense aromas (excellent acceptability)
Structural		
Crunchiness	Sensory attributes associated with firmness, fractureability, and density	0 No crunchy (not acceptable); 5 Moderate crunchy (limit acceptability); 9 Intense crunchy (excellent acceptability)
Dehydration	Dehydration of tissues	0 No Dehydration (excellent acceptability); 5 Moderate Dehydration (limit acceptability); 9 Severe Dehydration (not acceptable)

**Table 3:** Chemical characterization of coffee silverskin extract (CSE).

Total Bioactive Compounds			
TPC (mg GAE 100 mL^−1^)	TFC (mg ECE 100 mL ^−1^)		TTC (mg TAE 100 mL^−1^)
234.74	18.07		2.94
**Phenolic Acids (mg 100 mL^−1^)**			
Chlorogenic acid		Caffeic Acid	
33.99		15.21	
**Antioxidant Assays (mmolTE 100 mL^−1^)**			
ABTS	DPPH		FRAP
33.42	21.99		5.75

Abbreviations: TPC, Total Phenolic Compounds; TFC, Total Flavonoid Compounds; TTC, Total Tannin Compounds; ABTS, 2,2′-Azino-bis(3-ethylbenzothiazoline-6-sulfonic acid); DPPH, 2,2-Diphenyl-1-picrylhydrazyl; FRAP, Ferric Reducing Antioxidant Power; GAE, gallic acid equivalent; ECE, Epicatechin Equivalent; TAE, Tannic Acid Equivalent; TE, Trolox Equivalent.

**Table 4:** Colorimetric values of fresh-cut fennel slices during the storage period.

Parameter	Sample	Time (Days)				Sign.
		0	3	7	14	
L*	CTR	70.27 ± 2.10 ^A^	68.90 ± 2.69 ^bAB^	69.65 ± 1.94 ^bA^	66.55 ± 1.41 ^bB^	*
	AA	70.42 ± 1.18 ^B^	73.64 ± 2.25 ^aA^	73.08 ± 2.07 ^aA^	74.36 ± 2.49 ^aA^	*
	CS5	73.84 ± 4.59	73.48 ± 2.65 ^a^	73.94 ± 2.74 ^a^	75.80 ± 3.94 ^a^	n.s.
	CS10	70.15± 1.96 ^B^	74.85 ± 2.25 ^aA^	74.91 ± 2.10 ^aA^	75.27 ± 2.02 ^aA^	*
Sign.		n.s.	**	**	**	
a*	CTR	−1.1 ± 0.06 ^bC^	−0.57 ± 0.04 ^abB^	0.29 ± 0.04 ^aA^	0.39 ± 0.02 ^aA^	**
	AA	−1.06 ± 0.05 ^bB^	−0.91 ± 0.05 ^aB^	−0.16 ± 0.02 ^bA^	−0.13 ± 0.07 ^bA^	**
	CS5	−0.74 ± 0.04 ^a^	−0.59 ± 0.09 ^ab^	−0.66 ± 0.05 ^c^	−0.71 ± 0.04 ^c^	n.s.
	CS10	−0.51 ± 0.04 ^a^	−0.34 ± 0.02 ^b^	−0.38 ± 0.09 ^ab^	−0.59 ± 0.05 ^c^	n.s.
Sign.		*	*	**	**	
b*	CTR	11.82 ± 3.37	11.47 ± 2.90	10.93 ± 2.33 ^ab^	12.35 ± 2.64	n.s.
	AA	11.43 ± 2.91	10.66 ± 2.95	10.99 ± 2.32 ^a^	12.32 ± 3.23	n.s.
	CS5	11.79 ± 2.39	11.66 ± 2.83	10.84 ± 2.41^ab^	10.59 ± 3.83	n.s.
	CS10	11.61 ± 2.71	8.24 ± 4.44	9.00 ± 1.99 ^b^	11.62 ± 3.32	n.s.
Sign.		n.s.	n.s.	*	n.s.	

Small letters indicate significant differences between columns, and capital letters indicate significant differences between rows, as assessed by Tukey’s post hoc test. Abbreviations: **, significance at *p* < 0.01; *, significance at *p* < 0.05; n.s., not significant; CTR, control samples, AA, ascorbic acid dipping, CS5, coffee silverskin extract dipping (5%), CS10 coffee silverskin extract dipping (10%).

**Table 5:** Trends of malic, oxalic, and citric acids during the storage of fresh-cut fennel slices.

Parameter	Sample	Time (Days)				Sign.
		0	3	7	14	
Malic acid (mg 100 g^−1^)	CTR	165.11 ± 0.91 ^bB^	174.88 ± 1.88 ^bA^	165.08 ± 0.94 ^bB^	152.34 ± 0.56 ^cC^	**
	AA	166.55 ± 1.37 ^bB^	182.76 ± 0.94 ^aA^	171.47 ± 1.88 ^bB^	171.02 ± 2.62 ^bB^	**
	CS5	199.39 ± 3.60 ^aA^	183.92 ± 1.30 ^aB^	182.84 ± 3.38 ^aB^	178.92 ± 1.00 ^aB^	**
	CS10	192.13 ± 3.14 ^a^	180.14 ± 0.21 ^a^	180.98 ± 1.64 ^a^	179.32 ± 0.94 ^a^	**
	Sign	**	**	**	**	
Oxalic acid (mg 100 g^−1^)	CTR	150.28 ± 1.82 ^abB^	140.51 ± 0.04 ^Cb^	169.3 ± 4.92 ^abA^	164.84 ± 9.14 ^Ba^	*
	AA	156.13 ± 7.42 ^ab^	147.03 ± 5.36 ^c^	145.58 ± 7.17 ^c^	138.00 ± 3.84 ^c^	n.s.
	CS5	160.15 ± 3.77 ^ab^	158.88 ± 2.02 ^b^	155.57 ± 3.76 ^bc^	161.22 ± 1.23 ^b^	n.s.
	CS10	187.44 ± 13.62 ^a^	174.14 ± 0.93 ^a^	179.58 ± 0.45 ^a^	192.9 ± 0.38 ^a^	n.s.
	Sign	*	**	**	**	
Citric acid (mg 100 g^−1^)	CTR	165.34 ± 2.11 ^aB^	162.9 ± 3.28 ^Aa^	147.22 ± 0.16 ^bB^	117.59 ± 4.17 ^Ac^	**
	AA	146.6 ± 8.28 ^aB^	122.6 ± 6.16 ^abB^	101.85 ± 0.41 ^dc^	103.47 ± 7.84 ^aB^	**
	CS5	186.57 ± 1.95 ^aA^	181.97 ± 5.77 ^Aa^	164.27 ± 0.94 ^abB^	160.29 ± 0.24 ^bB^	**
	CS10	195.24 ± 5.63 ^Aa^	180.69 ± 5.28 ^aB^	187.81 ± 14.83 ^aB^	173.57 ± 3.87 ^bB^	n.s.
	Sign	**	**	**	**	

Small letters indicate significant differences between columns, and capital letters indicate significant differences between rows, as assessed by Tukey’s post hoc test. Abbreviations: **, significance at *p* < 0.01; *, significance at *p* < 0.05; n.s., not significant; CTR, control samples; AA, ascorbic acid dipping; CS5, coffee silverskin extract dipping (5%); CS10 coffee silverskin extract dipping (10%).

**Table 6:** TBC and yeast during the storage of fresh-cut fennel slices (Log10 CFU g^−1^).

Parameter	Sample	Time of Storage				Sign.
		0	3	7	14	
TBC	CTR	3.11 ± 0.02 ^D^	4.83 ± 0.11 ^C^	5.98 ± 0.18 ^Ba^	7.01 ± 0.02 ^Aa^	**
	AA	2.99 ± 0.16 ^D^	4.79 ± 0.08 ^C^	5.28 ± 0.03 ^Ba^	6.49 ± 0.03 ^Aab^	**
	CS5	3.04 ± 0.12 ^D^	4.77 ± 0.21 ^C^	5.33 ± 0.09 ^Ba^	6.36 ± 0.08 ^Ab^	**
	CS10	2.95 ± 0.07 ^C^	5.04 ± 0.22 ^B^	4.84 ± 0.12 ^Bb^	6.37 ± 0.11 ^Ab^	**
Sign.		n.s.	n.s.	**	*	
Yeast	CTR	0.00 ± 0.00 ^C^	1.79 ± 0.17 ^B^	1.89 ± 0.17 ^B^	2.84 ± 0.34 ^A^	**
	AA	0.00 ± 0.00 ^C^	1.95 ± 0.24 ^B^	1.86 ± 0.28 ^B^	2.85 ± 0.21 ^A^	**
	CS5	0.00 ± 0.00 ^C^	1.70 ± 0.01 ^B^	1.96 ± 0.24 ^B^	2.20 ± 0.71 ^A^	**
	CS10	0.00 ± 0.00 ^B^	1.80 ± 0.17 ^A^	1.86 ± 0.28 ^A^	2.35 ± 0.92 ^A^	**
Sign.		n.s.	n.s.	n.s.	n.s.	

Small letters indicate significant differences between columns, and capital letters indicate significant differences between rows, as assessed by Tukey’s post hoc test. Abbreviations: TBC, Total Bacterial Count; CTR, control samples; AA, ascorbic acid dipping; CS5, coffee silverskin extract dipping (5%); CS10 coffee silverskin extract dipping (10%). **, significance at *p* < 0.01; *, significance at *p* < 0.05; n.s., not significant.

## Data Availability

The original contributions presented in the study are included in the article. Further inquiries can be directed to the corresponding authors.
